# Entry of sapelovirus into IPEC-J2 cells is dependent on caveolae-mediated endocytosis

**DOI:** 10.1186/s12985-019-1144-6

**Published:** 2019-03-25

**Authors:** Tingting Zhao, Li Cui, Xiangqian Yu, Zhonghai Zhang, Xiaojuan Shen, Xiuguo Hua

**Affiliations:** 1 Shanghai Key Laboratory of Veterinary Biotechnology, School of Agriculture and Biology, Shanghai Jiao Tong University, 800 Dongchuan Road, Shanghai, China; 2 Shanghai Pudong New Area Center for Animal Disease Control and Prevention, Shanghai, 200136 China

**Keywords:** Endocytosis, Caveolin, Sapelovirus, Entry

## Abstract

**Background:**

Porcine sapelovirus (PSV), a species of the genus Sapelovirus within the family Picornaviridae, are a significant cause of enteritis, pneumonia, polioencephalomyelitis and reproductive disorders in pigs. However, the life cycle of PSV on the molecular level is largely unknown.

**Methods:**

Here, we used chemical inhibitors, RNA interference, and overexpression of dominant negative (DN) mutant plasmids to verify the roles of distinct endocytic pathways involved in PSV entry into porcine small intestinal epithelial cell line (IPEC-J2).

**Results:**

Our experiments indicated that PSV infection was inhibited when cells were pre-treated with NH_4_Cl or chloroquine. Inhibitors nystatin, methyl-β-cyclodextrin, dynasore and wortmannin dramatically reduced PSV entry efficiency, whereas the inhibitors chlorpromazine and EIPA had no effect. Furthermore, overexpression caveolin DN mutant and siRNA against caveolin also decreased virus titers and VP1 protein synthesis, whereas overexpression EPS15 DN mutant and siRNA against EPS15 did not reduce virus infection.

**Conclusions:**

Our findings suggest that PSV entry into IPEC-J2 cells depends on caveolae/lipid raft mediated-endocytosis, that is pH-dependent and requires dynamin and PI3K but is independent of clathrin and macropinocytosis.

## Background

Viruses generally enter cells via receptor-mediated endocytosis. The well-characterized clathrin-mediated endocytosis (CME) is the commonly used endocytic pathways for internalization of ligands, such as transferrin and epidermal growth factor [1], and many viruses, including type C foot-and-mouth disease virus [2] and echovirus 7 [3]. CME involves internalization of virus to be recruited to the plasma membrane through the formation of a clathrin coat [4]. The generated coated vesicles then deliver the virus particles into peripheral early endosomes, late endosomes and lysosomes.

Additionally, cells employ a number of alternative endocytic pathways resulting in processing outside the traditional CME. Among them, caveolae/raft dependent endocytosis is another well-characterized pathway. Caveolae, flask-shaped invaginations characterized by enrichment of caveolin-1 protein, could be used by cells to achieve rapid trafficking of external cargo to the endoplasmic reticulum or Golgi [5]. Caveolae are exploited by the simian virus 40 for trafficking to the endoplasmic reticulum independent of CME [6]. Echovirus, a nonenveloped RNA virus, enters into caco-2 cells via large, membranous, non-clathrin, non-caveolin-coated structures for distinct processing that depends on dynamin and cholesterol [7]. Murine norovirus-1 can also infect cells through non-clathrin and non-caveolae pathways [8]. Another mode of virus entry is by constitutive or induced macropinocytosis [9, 10], which depends on small GTPases of the Rho family and actin remodeling to promote formation of cell surface extrusions [11, 12].

Porcine sapelovirus (PSV) is a single-stranded, non-enveloped RNA virus, belonging to the genus of sapelovirus in the family Picornaviridae, and it is strongly associated with acute diarrhea, polioencephalomyelitis, pneumonia and reproductive disorders [13, 14]. PSV was first isolated from diarrheal pigs in China [13] and from wild boar in Japanese [15] in 2011 and was subsequently detected in pig diarrhea fecal samples throughout Spain, Korea, Brazil, and the Americas [14, 16–18], causing high morbidity and case fatality rate in the USA [14]. At present, researches on PSV are mainly about genomic characterization [19–21] and epidemiology [22]. Although α2,3-linked sialic acid on GD1a as a PSV receptor in LLC-PK1 cells has been found [22], research on the mechanism underlying the pathogenesis, replication, and entry of PSV has not yet been well established.

Different picornaviruses use a variety of entry routes into host cells, including CME, caveolae, and lipid rafts [23, 24]. In the current study, we addressed PSV entry into porcine small intestinal epithelial cell (IPEC-J2) by systematically perturbing the function of various cellular key factors involved in the known endocytic mechanisms using chemical inhibitors, siRNA silencing, and overexpression of dominant negative (DN) mutants of caveolin-1, Eps15 and dynamin-2. Our results suggested that PSV entry was caveolin-, lipid raft-, and dynamin-dependent and did not involve the clathrin and macropinocytosis pathway. Additionally, the viruses underwent slow acid-dependent penetration.

## Methods

### Cells and virus

IPEC-J2 cells were cultured in RPMI 1640 medium (Gibco, USA), supplemented with 10% fetal bovine serum (FBS, Gibco) in 5% CO_2_ at 37 °C. The PSV (csh) strain was isolated and preserved in our laboratory [13]. Mouse polyclonal anti-PSV VP1 antibody was generated by our laboratory.

### Inhibitors and cell viability assay

Inhibitors chlorpromazine (CPZ), ammonium chloride (NH_4_Cl), chloroquine (CQ), methyl-β-cyclodextrin (MβCD), nystatin, dynasore, 5-(N-ethyl-N-isopropyl) amiloride (EIPA), and wortmannin were purchased from Sigma, dissolved in water or DMSO and preserved in − 80 °C. Alexa Fluor 594-conjugated cholera toxin B (CTB) and Alexa Fluor 568-conjugated transferrin (Tfn) were purchased from Invitrogen (Carlsbad, CA, United States). Cell viability upon inhibitor treatment was assessed by employing the cell counting kit-8 (CCK-8, Beyotime Biotechnology, Shanghai, China). Briefly, cells were seeded in 96-well cell culture plates and subsequently treated with drugs at different concentrations for 24 h. After incubation with 10 μl CCK-8 for 2 h at 37 °C, the data of absorbance at a wavelength of 450 nm were collected.

### Cell infection and drug treatments

IPEC-J2 cell monolayers were grown in 24-well plates. After washing with DPBS for three times, the cells were pre-treated with inhibitors at the indicated concentrations for 1 h at 37 °C. For virus entry and replication assay, PSV was then added (MOI = 0.5). After incubation for 1 h, the unbound virus was removed by three washes with DPBS, and fresh medium was added to the cells. At 24 hpi, cells were lysed for virus titers determined by TCID_50_ or VP1 protein expression levels assays detected by western blot.

### Plasmids and siRNA transfection

Small interfering RNAs (siRNAs) against *Sus scrofa* clathrin heavy chain (siCHC-1, GCUCCAGAACCUGGGUAUATT; siCHC-2, GGAAGGAAAUGCAGAAGAATT), caveolin-1 (siCav, GCAAUAUCCGCAUCAACAUTT) and negative control (siNC, 5′-UUCUCCGAACGUGUCACGUTT-3′) were synthesized by GenePharma (Shanghai, China). IPEC-J2 cells were seeded on 24-well plates and transfected with siRNAs using lipofectamine 6000 (Beyotime Biotechnology) according to the manufacturer’s instructions. The knockdown efficiencies were quantified by RT-qPCR.

Plasmid expressing GFP-tagged Eps15 (WT), Eps15 (EpsΔ95/295), caveolin 1 wild type (Cav WT), caveolin 1 DN mutant (Y14F), dynamin-2 (WT) and dynamin-2 (K44A) were constructed by our laboratory and sequenced by Sangon (Shanghai, China). To determinate the infectivity of PSV in cells transfected with WT or DN mutant, IPEC-J2 cells grown on 24-well plates were fist transfected with 0.5 μg of plasmids for 24 h. Cells were then infected with PSV (MOI = 0.5), and virus replication was detected with western blot.

### RT-qPCR and western blot

After siRNA transfection for 30 h, cells were lysed and total RNA was extracted using TRIzol (Invitrogen). The mRNA levels of clathrin heavy chains (CHC) and caveolin-1 were checked by RT-qPCR. RT-qPCR was conducted with SYBR green master mix on an ABI 7500 Real-Time PCR System and 7500 System Software (Applied Biosystems, Alameda, CA, USA). For western blot analysis, cells were lysed in RIPA lysis buffer. After being separated by SDS-PAGE, the proteins were electrotransferred onto PVDF membranes and then immunoblotted with mouse anti-PSV VP1 antibody (1:1000) and anti-mouse secondary antibodies conjugated to HRP (1:10,000). α-tubulin was used as a loading control. Finally, bands were developed with ECL prime western blot detection reagent (GE Healthcare), and then quantified with Image Pro-Plus software.

### Tfn and CTB uptake assays

PK-15 cells seeded in 12-well plates with coverslips were left untreated or pretreated with indicated inhibitors for 1 h, and incubated with 50 μg/ml Alexa Fluor 568-conjugated Tfn or 10 μg/ml Alexa Fluor 594 conjugated-CTB at 37 °C for 60 min. Then, cells were washed with cold PBS for three times, fixed with cold 0.4% paraformaldehyde. Cell nuclei were stained with DAPI and cells were observed by confocal microscopy.

### Virus titration

Inhibitor treated and mock-treated IPEC-J2 cells infected with PSV were harvested at 24 h post-infection through freezing and thawing for three times, and then centrifuged to remove cell debris. Confluent cell monolayers in 96-well cell culture plates were incubated at 37 °C for 1 h with 10-fold serial dilutions of collected virus (100 μl/well). About 4–5 days later, cytopathic effect was recorded and virus titers were calculated using the Reed-Muench method and recorded as TCID_50_/100 μl.

### Statistical analysis

Data are presented as means ± SD for two independent experiments. All statistical analyses were performed using two-tailed student’s t-tests or one-way analysis of variance and Tukey post-hoc in GraphPad Prism. *P* < 0.05 was considered to be statistically significant.

## Results

### PSV infection of IPEC-J2 cells requires active endosomal acidification

**Fig. 1:**
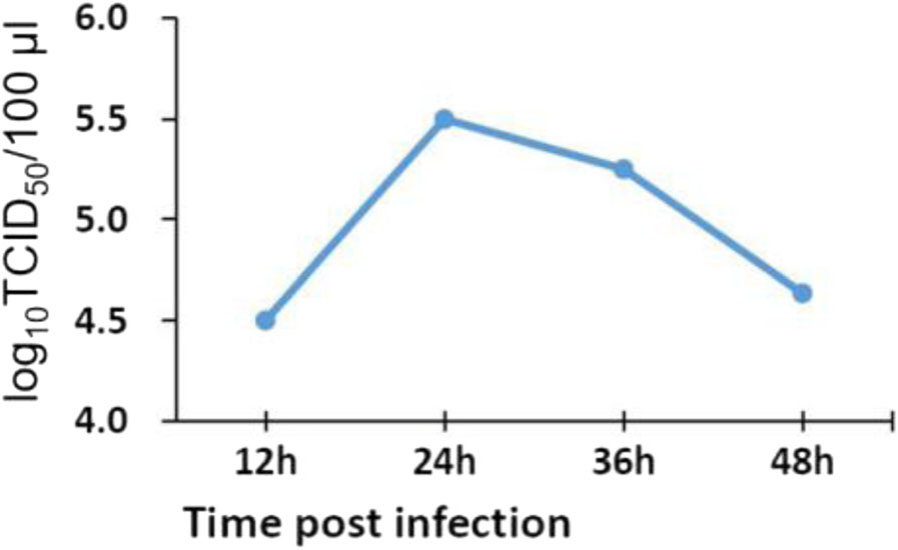
Endosomal acidification is required for PSV infection of IPEC-J2 cells. **a** and **b** IPEC-J2 cells were mock-treated or pre-treated with different concentrations of NH_4_Cl or chloroquine (CQ) at 37 °C for 1 h, and then the cells were inoculated with PSV (MOI of 0.5). At 24 hpi, infected cells were collected and virus titers were determined. The horizontal line shows results of subtoxic concentrations of NH_4_Cl or CQ on cells as determined by cell viability assay. Data are presented as means ± SD. The error bars indicate the standard deviations of triplicate samples from one of two independent experiments. **c** PSV protein synthesis was suppressed by pretreatment NH_4_Cl or CQ and in a concentration-dependent manner. At 24 h after virus infection, cell lysates were collected and the expression level of the viral major VP1 protein was measured by western blotting. ^*^P < 0.05; ^**^P < 0.01

To determine the effect of endosomal acidification on PSV infectivity, we exposed IPEC-J2 cells to increasing concentrations of CQ and NH_4_Cl for 60 min. The cells were then exposed to PSV at an MOI of 0.5 for 24 h for titer measurement and VP1 protein synthesis detection. NH_4_Cl and CQ, both lysosomotropic weak bases, inhibit endosomal acidification. Data from cell viability assays determined the subtoxic concentrations of the drugs (Fig. 1a and b). Compared to mock-treated cells (6.38 log_10_TCID_50_/100 μl or 6.22 log_10_TCID_50_/100 μl), the virus titer from cells treated with 5 mM NH_4_Cl or 10 μM CQ was 5.57 log_10_TCID_50_/100 μl or 5.38 log_10_TCID_50_/100 μl, respectively (Fig. 1a and b). Likewise, viral VP1 protein synthesis was also diminished by NH_4_Cl and CQ in a concentration-dependent manner (Fig. 1c). Together, these findings suggest that PSV enters IPEC-J2 cells uses a pH-dependent and, therefore, most likely an endosomal cell entry pathway.

### PSV entry and infection does not require clathrin

**Fig. 2:**
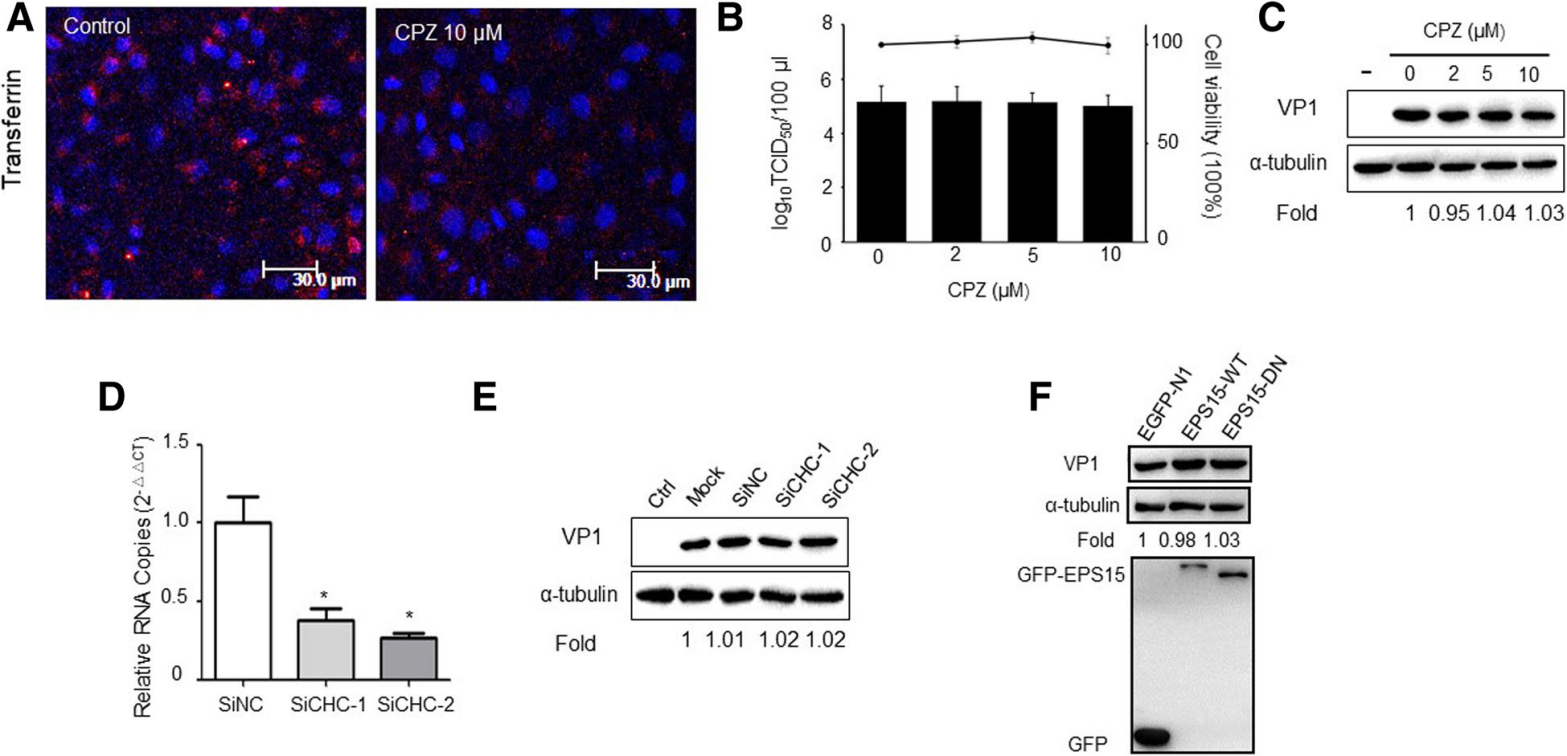
PSV entry and infection are independent of clathrin-mediated endocytic pathway. **a** The effect of CPZ (10 μM) on transferrin uptake in PK-15 cells was observed using confocal microscopy. **b**-**c** Viral yield assays in CPZ or mock-treated IPEC-J2 cells. Cells were pretreated with CPZ at the subtoxic concentrations (**b**, horizontal line) and then infected with PSV in the presence of CPZ for 1 h. At 24 hpi, cells were collected and the progeny virus titer was determined by TCID_50_ and the expression level of viral VP1 proteins was analyzed by western blot. **d** The effect of siRNA against clathrin heavy chains (CHC) was determined by RT-qPCR. **e**-**f** IPEC-J2 cells were transfected with siRNA against negative control (siNC), or two siRNAs targeting CHC or cells were transfected with EGFP, EGFP-tagged wild-type Eps15 (Eps15wt), or DN mutant Eps15 (EpsΔ95/295). At indicated time after transfection, cells were infected with PSV and processed for western blot. Data are presented as means ± SD. The error bars indicate the standard deviations of triplicate samples from one of two independent experiments. Bars, 30 μm. ^*^P < 0.05; ^**^P < 0.01

CPZ is a commonly used chemical inhibitor for interfering with CME through preventing the assembly of clathrin lattices on endosomal membranes and the assembly of clathrin coated pits at the cell surface [25]. To examine the role of clathrin-dependent endocytic pathways in PSV entry and infection of IPEC-J2 cells, we first tested the effect of CPZ on transferrin uptake, which is a model for the CME pathway. As shown in Fig. 2a, when treated with10 μM CPZ, the signal intensity of fluorescently labeled transferrin was obviously reduced. Then we treated IPEC-J2 cells with increasing concentrations of CPZ at subtoxic concentrations (Fig. 2b). However, PSV virus titers or VP1 protein synthesis did not appear to be reduced in the presence of CPZ (Fig. 2b, c). We next tested the effect of knocking down CHC on PSV infection. The siRNA efficiency against CHC was examined by RT-qPCR (Fig. 2d). IPEC-J2 cells were then transfected with siRNA negative control, a non-targeting CHC, or a siRNA targeting against CHC. At 30 h after transfection, cells were infected with PSV (MOI = 0.5), and at 24 h post infection cell lysates were collected, and detected by western blot. The siRNA targeting CHC also did not reduce viral infection (Fig. 2e). To further confirm that PSV enters IPEC-J2 cells does not require CME, we transfected Eps15, which is crucial component of clathrin coated pits and interacts with adaptor protein 2 [26]. Similarly, no significant reduction in VP1 protein synthesis was observed with transfecting wild-type or DN mutant Eps15 (Fig. 2f). Collectively, these data indicate that CME may be not an essential pathway for PSV infection.

### Entry of PSV may be caveolae and cholesterol dependent

**Fig. 3:**
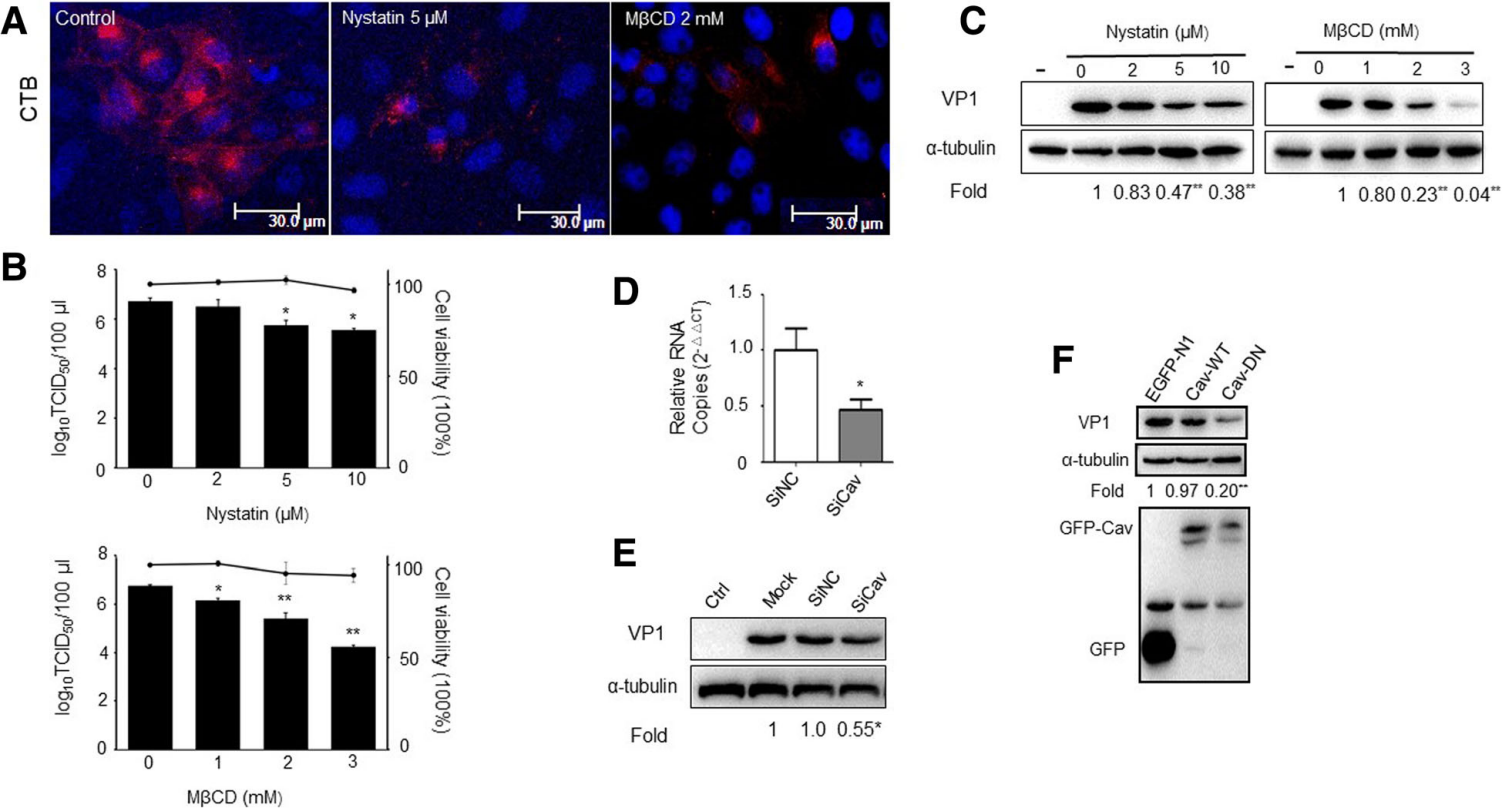
Caveola/raft-dependent endocytosis is required for PSV infection. **a** Cholera toxin B (CTB) uptake was blocked by nystatin or MβCD. PK-15 cells were mock treated or pretreated with nystatin or MβCD at indicated concentrations for 1 h, and then 10 μg/ml Alexa Fluor 594 conjugated-CTB was added and incubated at 37 °C for 60 min before observed using confocal microscopy. **b**-**c** Nystatin and MβCD reduced PSV infection. IPEC-J2 cells were pretreated with increasing concentrations of nystatin or MβCD for 1 h at 37 °C, and then cells were infected with PSV (MOI = 0.5). At 24 hpi, cells were lysed and virus titers were determined by TCID_50_ or viral VP1 expression levels were detected by western blot. The horizontal line shows the subtoxic concentrations of nystatin or MβCD on IPEC-J2 cells. **d** The effect of siRNA against caveolin-1 was determined by RT-qPCR. **e**-**f** Cells were transfected with siRNA against negative control (siNC), siRNA against caveolin-1 (siCav), or cells were transfected with EGFP, EGFP-tagged wild-type caveolin-1 (Cav-WT), caveolin 1 dominant negative mutant (Y14F) (Cav-DN). At 24 h after transfection, cells were infected with PSV, and virus VP1 protein levels were detected by western blot. Data are presented as means ± SD. The error bars indicate the standard deviations of triplicate samples from one of two independent experiments. Bars,30 μm. ^*^P < 0.05; ^**^P < 0.01

Caveolae are plasma membrane invaginations which are involved in numerous cellular processes including transport, signaling, and tumor suppression. The formation of caveolae depends on the expression of caveolin-1 (Cav1). Cav1 directly interacts with cholesterol of the plasma membrane [27] and depletion of the cholesterol makes caveolae flatten [28]. To investigate the possible involvement of caveolae/lipid rafts in PSV entry, we first tested the effect of cholesterol sequestrator nystatin and the caveolae-dependent endocytic inhibitor MβCD on CTB uptake assays. A significant reduction in the signal intensity of fluorescently labeled CTB was observed upon either 5 μM nystatin or 2 mM MβCD pretreatment, indicating a block in CTB uptake (Fig. 3a). Therefore, prior to PSV infection, we treated IPEC-J2 cells with increasing concentrations of nystatin and MβCD at subtoxic concentrations (Fig. 3b). We observed that 10 μM nystatin and 3 mM MβCD reduced virus titers by about 1 log_10_ and 2 log_10_, respectively, compared with the untreated controls (Fig. 3b). Similarly, at an MOI of 0.5, VP1 protein levels decreased by 53 and 62% in 5 μM nystatin and 10 μM nystatin pretreatment, respectively, and by 77 and 96% in 2 mM MβCD and 3 mM MβCD pretreatment (Fig. 3c).

Furthermore, cell perturbation assays using siCav were carried out to evaluate the role of caveolin in virus infection. The siRNA efficiency was first detected using RT-qPCR, and siCav could specifically decrease caveolin expression (Fig. 3d). Then, siNC or siCav was transfected into the IPEC-J2 cells. At 30 h after transfection, we performed PSV entry assays, and found that siCav specifically decreased viral VP1 protein expression approximately 45% (Fig. 3e). We also transfected IPEC-J2 cells with a dominant-negative form of the protein with a Y14F mutation, which has been reported to inhibit caveolae dependent endocytosis [29]. IPEC-J2 cells were transfected with either caveolin-1 WT or DN plasmid caveolin-1 Y14F as well as control vector before virus infection. We found that PSV VP1 protein synthesis was significantly decreased by approximately 80% in cells expressing caveolin-1 DN compared to cells transfected with empty vector (Fig. 3f). Together, the data suggest that PSV enters IPEC-J2 cells depend on caveolae and lipid raft.

### PSV enter IPEC-J2 cells requires dynamin

**Fig. 4:**
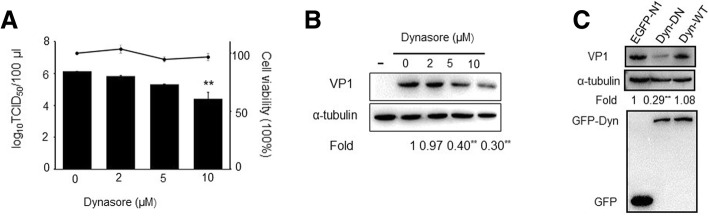
PSV infection occurs in a dynamin-dependent manner. **a**-**b** Effect of pretreatment of IPEC-J2 cells with increasing concentrations of dynasore on PSV titer as determined by TCID_50_ or virus VP1 protein synthesis as detected by western blot. The horizontal line shows the subtoxic concentrations of dynasore on IPEC-J2 cells. **c** IPEC-J2 cells were transfected with plasmids encoding wild-type dynamin-GFP (Dyn-WT) or the DN mutant dynamin (K44A) (Dyn-DN). At 30 h after transfection, cells were infected with PSV and quantified PSV VP1 protein expression was performed by western blot. Data are presented as means ± SD. The error bars indicate the standard deviations of triplicate samples from one of two independent experiments

Dynamin-2, a regulatory GTPase, is essential for the fission of caveolae from the plasma membrane [30]. Dynasore, a cell-permeable dynamin GTPase activity inhibitor, was used to detect the role of dynamin during PSV infection in this study. We observed about 2 log_10_ decrease in virus titer in cells pretreated with 10 μM dynasore compared with the untreated cells (Fig. 4a). Furthermore, dynasore reduced PSV VP1 protein synthesis by 60% at 5 μM, whereas at a concentration of 10 μM, dynasore reduced PSV VP1 protein synthesis by 70% (Fig. 4b). To further verify the possible involvement of dynamin in PSV entry, we transfected IPEC-J2 cells with plasmids encoding wild-type dynamin-GFP or the DN mutant dynamin (K44A) and found that expression of the DN mutant dynamin (K44A) could decrease PSV VP1 protein synthesis by approximately 71% (Fig. 4c), suggesting that PSV entry into IPEC-J2 cells require dynamin.

### PSV entry is macropinocytosis independent but requires phosphatidy-linositide 3-kinases (PI3K)

**Fig. 5:**
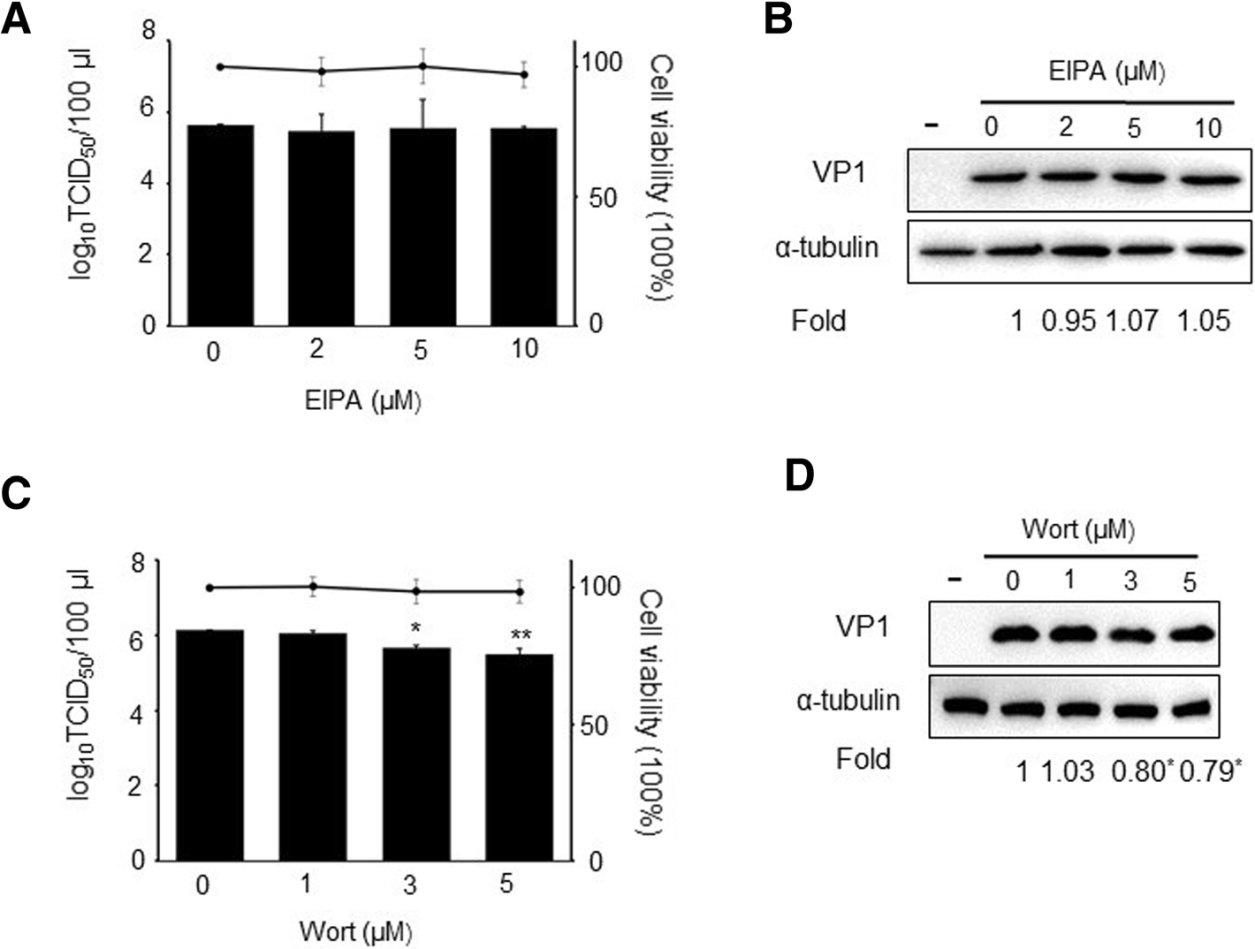
PSV entry is macropinocytosis independent but requires PI3K. **a**-**b** Cells were pretreated with EIPA at different concentrations for 1 h after virus was added. After 24 h, cells were collected for virus titer or VP1 protein synthesis detection. **c**-**d** Cells were treated with wortmannin the same as EIPA. The horizontal line shows the subtoxic concentrations of EIPA or wortmannin on IPEC-J2 cells. Data are presented as means ± SD. The error bars indicate the standard deviations of triplicate samples from one of two independent experiments. ^*^P < 0.05; ^**^P < 0.01

Macropinosome formation depends on Na^+^/H^+^ exchangers, and EIPA is an Na^+^/H^+^ antiport inhibitor, therefore, inhibits macropinocytosis-mediated endocytosis [12]. Wortmannin is a covalent inhibitor of PI3K, which is involved in multiple stages of macropinocytosis [31]. To investigate whether PSV entry involves macropinocytosis and PI3K, PSV was added to IPEC-J2 cells pretreated with EIPA or wortmannin. EIPA had no effect on PSV titer or VP1 protein synthesis at different concentrations, as indicated by TCID_50_ assays or western blot (Fig. 5a,b). However, 5 μM wortmannin decreased virus titer by about 1 log_10_ and decrease PSV VP1 protein synthesis by 21%, respectively (Fig. 5c, d). These findings suggested that PSV entry and replication does not depend on macropinocytosis, but requires P13K.

## Discussion

Virus internalization and entry into their target cells may use multiple pathways in different types of cells [31]. To investigate the possibility of PSV uses multiple endocytic pathways in IPEC-J2 cells, systematic approaches were used, including pharmacological inhibition, RNA interference, and overexpression of DN mutant plasmids. We show here that PSV does not depend on CME or macropinocytic endocytosis. Instead, PSV enters IPEC-J2 cells via a caveolae/lipid raft pathway requiring dynamin and a low-pH environment. Understanding the trafficking pathway that PSV undergoes not only provides improved understanding of viral biology but also is crucial for finding drug targets.

In general, pH changes are not essential for virus entry through plasma membranes by direct fusion, whereas a low pH environment is required when viruses usurp cellular endocytic pathways [32]. Virions are often exposed to the acidic milieu of endosomes merely within minutes following complete internalization. In some cases, acidic pH alone is insufficient to induce fusion for some viruses, including many mammalian reoviruses, and acid-dependent endosomal proteases mediated cleavages in viral proteins are essential for triggering the variation to the penetration-competent condition [33]. The weak lysosomotropic bases that diffuse into acidic endosomes increase the endosomal pH, leading to inhibition of virus infection by human rhinovirus and equine rhinitis A virus [34, 35]. Treatment with NH_4_Cl or CQ that disrupt cellular pH decreased PSV titer and VP1 protein synthesis in a concentration-dependent manner, indicating a pH-dependent uptake mechanism.

The clathrin-mediated endocytic route is the most commonly used endocytic pathways taken by viruses. It transports incoming virions together with their receptors into early and late endosomes. CME is characterized by the formation of heavily coated pits at the plasma membrane indentations and the formation of characteristic clathrin-coated vesicles [32, 36]. Here, we used CPZ, siRNA against EPS15 and EPS15 DN mutant fused to GFP to specifically block CME. Our results demonstrated that CPZ inhibited transferrin uptake, but did not decrease PSV titer or VP1 protein expression levels. Similarly, siRNA against EPS15 and EPS15 DN mutant transfection did not significantly alter PSV infection, indicating that PSV entry IPEC-J2 cells may be independent of CME.

Caveolae-mediated endocytosis is the major route of entry for foot-and-mouth disease virus, echovirus 1 [37] and other viruses [38, 39]. The feature of this pathway is its dependence on caveolin-1 in non-muscle cells [40]. Caveolae endocytosis requires dynamin, which is located in the neck of caveolae either constitutively in endothelial cells [41], or is recruited in response to specific signals [6]. Cells treated with caveolae-mediated-endocytosis inhibitors became resistant to PSV entry, and caveolin-1 knockout as well as caveolin DN mutant transfection also inhibited virus infection. Together, these data indicate that caveolae/ lipid raft-mediated endocytosis is likely the main pathway used by PSV to enter IPEC-J2 cells.

Dynamin conduces to membrane fission to generate endocytic vesicles and is required for many endocytic pathways. Endocytic pathways can be divided into subpathways that are dependent on dynamin, including CME, caveolae-mediated endocytosis, and clathrin-independent dynamin-mediated pathways, and those that are not dependent on dynamin, which included macropinocytosis, lipid raft-mediated endocytosis, and non-clathrin/non-caveolae endocytosis [11, 27, 42]. Our studies with the dynamin inhibitors dynasore and dynamin DN mutant indicate that PSV perhaps utilize a dynamin-mediated pathway.

Macropinocytosis is a transient, actin-dependent cellular process used by cells to internalize significant amounts of fluids and membrane [43]. The process requires actin polymerization but does not dependent on dynamin. It has been verified to be used by vaccinia virus [44] and measles virus [45]. As NHE is essential for the formation of macropinocytic protrusions, we used the Na^+^/H^+^ inhibitor EIPA to detect the role of macropinocytosis in PSV entry. Treatment with 10 μM EIPA did not reduce the virus titer or VP1 protein synthesis, suggesting that the entry of PSV into IPEC-J2 cells may be independent of macropinocytosis. Wortmannin could inhibit members of the polo-like kinase family and PI3K-related kinases, including mTOR, ATR and the catalytic subunit of DNA-dependent protein kinase [46–48]. Although our data imply that wortmannin could reduce PSV replication, the precise mechanism remains to be elucidated.

There are still some limitations in the present study. First, viruses enter different target cells may use different pathways. PSV could be cultivated in many cells, including pig cells (PK-15, IPEC-J2, IBRS-2 and LLC-PK) [13, 22] and human hepatocarcinoma cell line (PLC/PRF/5 and HepG2/C3a) [20]. We only used IPEC-J2 cells as the target cells and other alternative cell lines were beyond the scope of the current study. Second, the actin and microtubule cytoskeleton play crucial roles in endocytosis and intracellular trafficking, but the effect of inhibition of actin and microtubules on PSV infection was not performed. Third, specific effects of inhibitors treatment after PSV infection are unknown. Hence, further work is obligatory to solve those issues above in the future.

## Conclusions

In conclusion, we conducted a systematic research to clarify the entry mechanism of PSV in IPEC-J2 cells. Our data suggest that PSV enters cells perhaps through dynamin-, cholesterol-dependent, and caveolin-mediated endocytosis that requires low pH. Findings of this study may provide new insights into the biological characteristics of these RNA viruses and open new opportunities to develop novel therapeutic approaches.

## Abbreviations

Cav1: Caveolin-1
CCK-8: Cell counting kit-8
CHC: Clathrin heavy chains
CME: Clathrin-mediated endocytosis
CPZ: Chlorpromazine
CQ: Chloroquine
CTB: Conjugated cholera toxin B
DN: Dominant negative
EIPA: 5-(N-ethyl-N-isopropyl) amiloride
IPEC-J2: Porcine small intestinal epithelial cells
MβCD: Methyl-β-cyclodextrin
NH_4_Cl: Ammonium chloride
PSV: Sapelovirus
siRNAs: small interfering RNAs
